# Nectar secretion in a dry habitat: structure of the nectary in two endangered Mexican species of *Barkeria* (Orchidaceae)

**DOI:** 10.7717/peerj.11874

**Published:** 2021-08-02

**Authors:** Małgorzata Stpiczyńska, Magdalena Kamińska, Kevin L. Davies

**Affiliations:** 1 Faculty of Biology, Botanic Garden, University of Warsaw, Warsaw, Poland; 2 Department of Botany and Plant Physiology, University of Life Sciencies in Lublin, Lublin, Poland; 3 School of Earth and Ocean Sciences, Cardiff University, Cardiff, United Kingdom

**Keywords:** Barkeria, Orchidaceae, Histochemistry, Nectary, Structure, Nectar secretion, Drought-induced stress

## Abstract

*Barkeria scandens* and *B. whartoniana* are endangered, endemic taxa from Mexico. They are epiphytes adapted to dry habitats. Since these plants are xerophytic, their flowers were investigated for structural adaptations to nectar secretion. The flowers of both species are structurally similar, and contrary to most claims for the genus, have functional floral nectaries comprising a nectary chamber and a narrow tubular cuniculus. Nectar is present in both these structures, and contains sugars and lipid-like compounds. The nectary tissue is composed of a single-layered epidermis overlying 1–2 layers of subepidermal secretory parenchyma. The outer tangential wall of the epidermal cells is thick and multi-layered, whereas the cuticle, which often shows blistering, is lamellate and possesses micro-channels. Lipid-like material occurs both between the microfibrils of the cell wall and in the micro-channels. Robust secretory tissue, thick cell walls, and lipid-like nectar components limit nectar evaporation. Moreover, the rigidity of the nectary potentially makes it possible for red-flowered *B. scandens* to switch from entomophily to ornithophily.

## Introduction

The genus *Barkeria* Knowles & Westc. comprises 16–17 species (Pridgeon et al., 2010; Soto Arenas, 2005) that are distributed from Mexico to Central America along the Pacific coast. It represents a relatively young, monophyletic group belonging to the higher Epidendroid clade, is adapted to dry habitats, and has colonized Mexican dry forests (Angulo, Ruiz-Sanchez & Sosa, 2012; Halbinger, 1977; Salazar & Arguijo, 1993; Soto Arenas, 2005; Thien & Dressler, 1970; Withner, 1998).

*Barkeria scandens* (La Llave & Lex.) Dressler & Halb. is a rare, endangered endemic species that inhabits oak or pine-oak semi-dry forests in Guerrero and Oaxaca State, at altitudes of 1,800–2,700 m a.s.l. and an annual mean temperature of 18.38 °C (Angulo, Ruiz-Sanchez & Sosa, 2012). This is a rupicolous or epiphytic, small-to medium-sized plant that grows on *Clusia* (Clusiaceae), *Oreopanax* (Araliaceae), *Roupala* (Proteaceae) (Soto Arenas & Solano-Gómez, 2007) and twigs of *Randia* (Rubiaceae) (Pridgeon et al., 2010). Its flowers are long-lived, lasting up to 3 months. They are intense magenta in colour, with white markings on the labellum, and lack a fragrance perceptible to humans. In Michoacán, demand for cut flowers has resulted in the destruction of local populations of this species (Pridgeon et al., 2010).

*Barkeria whartoniana* (C. Schweinf.) Soto Arenas is also an endangered micro-endemic species occurring in Oaxaca State, in particular, in the ‘rock gardens’ of the Isthmus of Tehuantepec. This small-sized plant grows exclusively amongst sparse vegetation on calcareous outcrops (Pridgeon et al., 2010). It inhabits seasonal tropical dry forest or areas supporting xerophytic lowland vegetation, with an annual mean temperature of 25.25 °C (Angulo, Ruiz-Sanchez & Sosa, 2012). According to Segovia-Rivas et al. (2018), its populations are extremely small, the plants occupying an area of only ca. 26 km^2^. Soto Arenas (2005) described *B. whartoniana* as being predominantly rupicolous, but more recent reports indicate that it is epiphytic, growing on *Comocladia engleriana* (Anacardiaceae), *Neobuxbaumia scoparia* and *Neodawsonia* sp. (Cactaceae), as well as on *Beaucarnea* sp. (Asparagaceae) (Pérez García, 2013; Pridgeon et al., 2010; Segovia-Rivas et al., 2018). Pridgeon et al. (2010), however, state that this species starts life as an epiphyte, but may become detached from the host plant and, on landing on rocks, may develop into large lithophytic specimens. Currently, studies on the host preferences of this endangered species, and efforts to reintroduce it into its former habitats are under way (Segovia-Rivas et al., 2018). Its flowers are pale lilac, with dark red or maroon markings on the lip, and these, as in the previous species, lack fragrance.

In *Barkeria*, the labellum, which is a modified petal distinguished from the other petals and sepals by its larger size and ornamentation, is clawed and fused basally to the column. Soto Arenas & Solano-Gómez (2007) reported that the flowers of *B. scandens* are nectarless, but according to van der Pijl & Dodson (1966), the flowers of *B. lindleyana*, a species closely related to *B. scandens*, are pollinated in Costa Rica by the carpenter bee *Xylocopa tabaniformis*, and demonstrate pollination efficiency of ca. 20%. It was claimed that a single bee pollinated 150 of an estimated 700–800 flowers of this species in 2 days; behaviour that would suggest the presence of a reward. By contrast, nectar is considered to be absent in other species of *Barkeria*. We were not able to find further published information on nectar secretion in *B. whartoniana*, or on any of its pollinators, but *e.g.*, in *B. lindleyana*, insect pollinators must push their way between the labellum and the adpressed column. Consequently, small insects such as the solitary sweat bee *Auglochlora*, the orchid bee *Euglossa* and skipper butterflies (Hesperiidae) are not effective pollinators of this species. Although little is currently known about the pollinators of *B. scandens* and *B. whartoniana*, plants of the former species cultivated in the gardens of New Mexico, a considerable distance from their natural habitat, are commonly pollinated by the hummingbirds *Amazilia tzacatl* and *Hylocharis leucocollis* (Warford, 1993). *Barkeria scandens*, in particular, displays a degree of ornithophily, albeit in cultivation (Warford, 1993). This species may be truly entomophilous, but opportunistically ornithophilous when its natural insect pollinator is absent. However, this claim demands further investigation in its natural habitat. Information on reproduction in *Barkeria* and its relationships with pollinators may prove to be particularly important in those rare species, such as *B. whartoniana* and *B. scandens*, that face the threat of extinction. Angulo, Ruiz-Sanchez & Sosa (2012); propose that pollinator limitation may perhaps contribute toward the absence of *Barkeria* spp. from their predicted potential niches.

The presence of a floral reward in orchids, as in many other angiosperms, is an important factor in attracting pollinators. Nectar is the most common floral food-reward, its presence both increasing the number of visits by pollinators and improving reproductive success (Neiland & Wilcock, 1998). Nevertheless, it should be stressed that in Orchidaceae, in order to save resources and increase pollination success, deception is also common. For example, in certain European genera such as *e.g., Dactylorhiza*, *Anacamptis* and *Orchis* (Bell et al., 2009), as well as in some species of neotropical *Epidendrum e.g., E. fulgens* (Moreira, Fuhro & Isaias, 2008), the nectaries are devoid of nectar and are represented by floral spurs and a cuniculus, respectively. Furthermore, in these examples, the inner epidermis bears trichomes similar to those present in nectariferous species, and these trichomes probably provide tactile stimuli that deceive pollinators. This is congruent with results obtained by Cardoso-Gustavson et al. (2018) in a study on the evolution of nectar gain and loss in 27 species of *Epidendrum* subgenus *Amphyglottium*. These authors noted that species having cuniculi lined with smooth epidermis secrete nectar, whereas those with cuniculi lined with papillae or trichomes are nectarless.

In both investigated species of *Barkeria*, the nectary comprises a nectary chamber and cuniculus, as has been reported for other certain nectariferous representatives of Laeliinae (Stpiczyńska et al., 2018 and references therein). In view of the lack of information and the inconsistent data on nectar secretion in *Barkeria*, we aim to verify the presence of floral nectariferous tissue in *B. scandens* and *B. whartoniana* and, should this be present, to study its structure and adaptations for nectar secretion in a dry habitat. For the purpose of this study, we selected two species belonging to the same phylogenetic lineage (Soto Arenas, 2005).

## Materials & methods

Plants of *Barkeria scandens* (Lex.) Dressler & Halb. and *Barkeria whartoniana* (C. Schweinf.) Soto Arenas used in this study were cultivated at Swansea Botanical Complex, UK. They were purchased as seedlings from Richard Warren (Equatorial Plant Company, 7 Gray Lane, Barnard Castle Co. Durham. UK.) on 5.xi.1995 and on 5.ix.1997, and bear the Swansea accession numbers S19950035 and S19970005, respectively. The study material was supplemented by flowers of *B. whartoniana* obtained from the living collection of Maria and Grzegorz Garbuz, Zamość, Poland. These plants were purchased in 2019 from Orchids & More, Mayerbacherstraße 94, 85,737 Ismaning, Germany. Spirit-preserved voucher material was deposited at the herbarium of Warsaw Botanic Garden, Poland, under the following accession numbers: WABG002668 (*B. scandens*), WABG002669 (*B. wharthoniana* from Swansea Botanical Complex) and WABG002670 (*B. wharthoniana* from Zamość).

The position of the nectary in longitudinally hand-sectioned flowers of both investigated species and the presence or absence of nectar were determined by means of a Nikon SMZ100 stereomicroscope. Flowers at the bud stage, about 2 days before opening (three buds for *B.whartoniana* and one bud for *B.scandens*), and at the 1st day of anthesis (three flowers for each species), were checked for nectar. Owing to the limited number of *B. whartoniana* flowers available and the small volumes of secretion produced, we were able to use refractometry (RL-4 PZO) only on a single 20 µL sample for this species. Nectary tissue was subsequently examined using light microscopy (LM), fluorescence microscopy (FM), scanning electron microscopy (SEM) and transmission electron microscopy (TEM).

Histochemical investigations were performed on hand-sectioned living or fixed material, and on semi-thin sections of fixed material. Hand-sectioned tissue (fresh or fixed in 70% ethanol) was examined using Nomarski differential interference microscopy (NDIM). The presence and distribution of lipids and starch were established by treating the tissue with a saturated ethanolic solution of Sudan IV or with an aqueous solution of Lugol’s iodine solution (IKI), respectively, followed by examination using a Nikon Eclipse Ni-U light microscope. Autofluorescence of nectary tissue and nectar was investigated by means of a Nikon Eclipse Ni-U microscope equipped with a Prior 200 W lamp (Prior Scientific Instruments Ltd., Cambridge, United Kingdom) and UV-2B cube filter 355/50 (330–380 nm excitation filter; a 400 nm (LP) dichroic mirror and a 435 nm (LP) barrier filter). Volatiles were detected by treating sections with 0.1% (w/v) aqueous neutral red solution for 20 min and observing them under UV-B light (Kirk, 1970). Sections stained with auramine O were examined under blue light for detail of the cell wall and cuticle using a FITC-Nikon cube filter (Gahan, 1984). Micrometry and photomicrography were accomplished by means of a Nikon Eclipse Ni-U microscope equipped with NIS-Elements BR software and DS-Ri2 camera.

For semi-thin sections and TEM observations, pieces of nectary tissue measuring ca. 3 mm^3^ were excised and fixed in 2.5% (v/v) glutaraldehyde/4% (v/v) formaldehyde in sodium cacodylate buffer (pH 7.0; 0.1 M) for 2 h at 4 °C, washed three times in the same buffer and post-fixed in 1.5 % (w/v) osmium tetroxide solution for 2 h at 0 °C. The fixed material was then dehydrated using a graded ethanol and acetone series, and infiltrated and embedded in Spurr’s resin (Low Viscosity Embedding Kit, medium grade, Sigma-Aldrich, St. Louis, MI, USA) followed by polymerization at 60 °C. Semi-thin sections (0.9-1.0 µm thick) were prepared for general histology of the nectary, and stained with a 1:1 solution of 1% (w/v) aqueous methylene blue: 1% (w/v) aqueous azure II (MB/AII) for 5-7 min at 60°C. The periodic acid-Schiff (PAS) reaction was also employed to detect the presence of insoluble polysaccharides (Jensen, 1962), whereas an ethanolic solution of Sudan Black B (SBB) was used for the detection of lipids.

For transmission electron microscopy (TEM), sections were cut at 70 nm using a Reichert Ultracut-S ultramicrotome and a glass knife, stained with uranyl acetate and lead citrate (Reynolds, 1963) and examined using a JEOL transmission electron microscope, at an accelerating voltage of 80 kV. A high-resolution digital camera (CCD MORADA, SiS-Olympus, Germany) was used to capture TEM images.

For scanning electron microscopy (SEM), fixed flowers were cut longitudinally to expose the entire surface of the nectary, or cut transversely, dehydrated in a graded acetone series and subjected to critical-point drying using liquid CO_2_. The preparations were then sputter-coated with gold and examined using a LEO 1,430VP (Zeiss) scanning electron microscope, at an accelerating voltage of 3.00 kV

## Results

**Figure 1:**
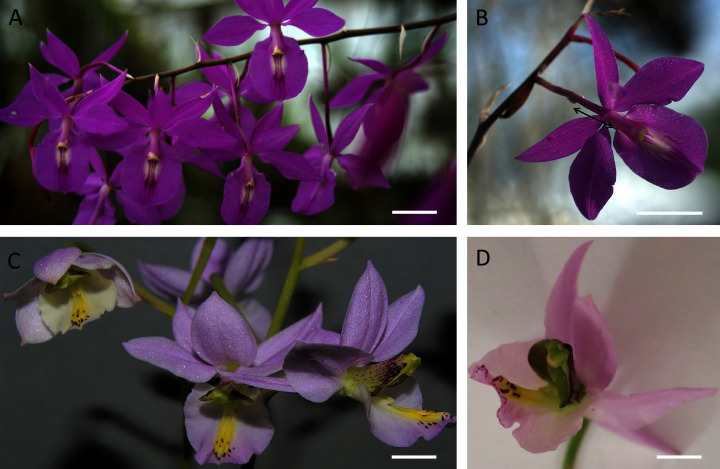
Inflorescence and flowers of *B. scandens* (A, B) and *B. whartoniana* (C, D). In B, black double ended arrow indicates position of the nectary cuniculus, which is the same in both species. Scale bars =1 cm.

**Figure 2:**
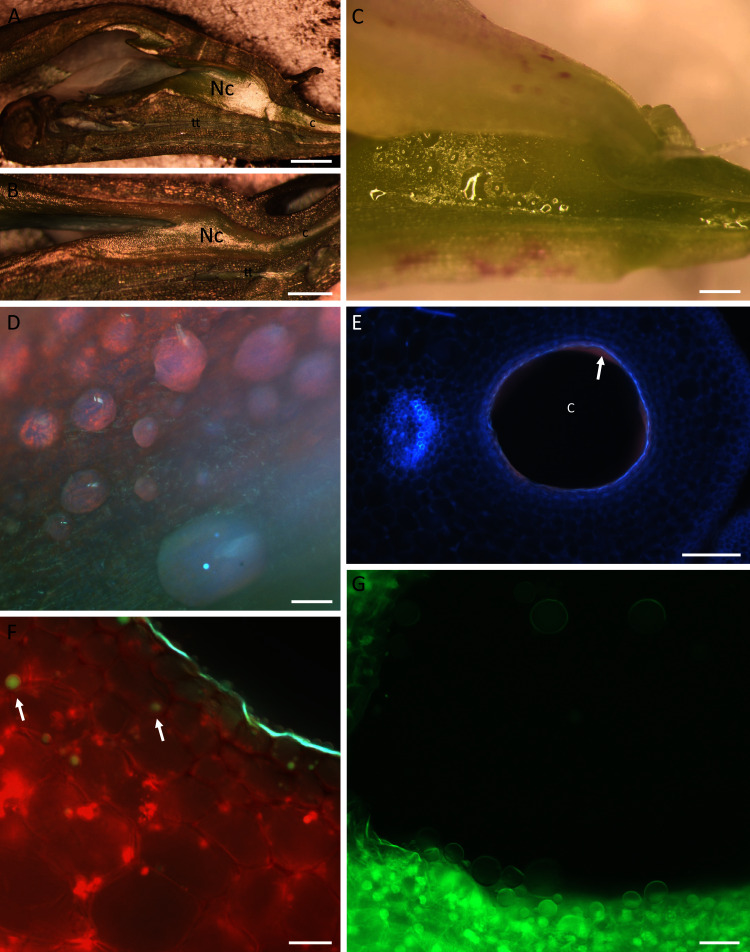
Nectaries and nectar of *B.whartoniana* (A, C–G) and *B.scandens* (B). (A, B) Position of the nectary chamber and cuniculus in relation to that of the column, transmitting tract and labellum; scale bar = 1 mm. (C) Nectar droplets in the nectary chamber at the late bud stage; longitudinal section, illuminated with natural light; scale bar = 0.5 mm. (D) Droplets of nectar in the nectary chamber of a flower at anthesis, illuminated with UV light; scale bar = 100 µm. (E) Transverse section of the cuniculus. Note the red autofluorescence of the nectar (arrow); scale bar = 100 µm. (F) Epidermal and subepidermal parenchyma cells stained with neutral red and illuminated with UV light. Note the blue-green and yellow-green fluorescence of the cuticle and drops of secretion, respectively (arrows); scale bar = 20 µm. (G) Nectar drops within the cuniculus stained with auramine O; scale bar = 50 µm. c–cuniculus, Nc-nectary chamber, tt-transmitting tract.

**Figure 3:**
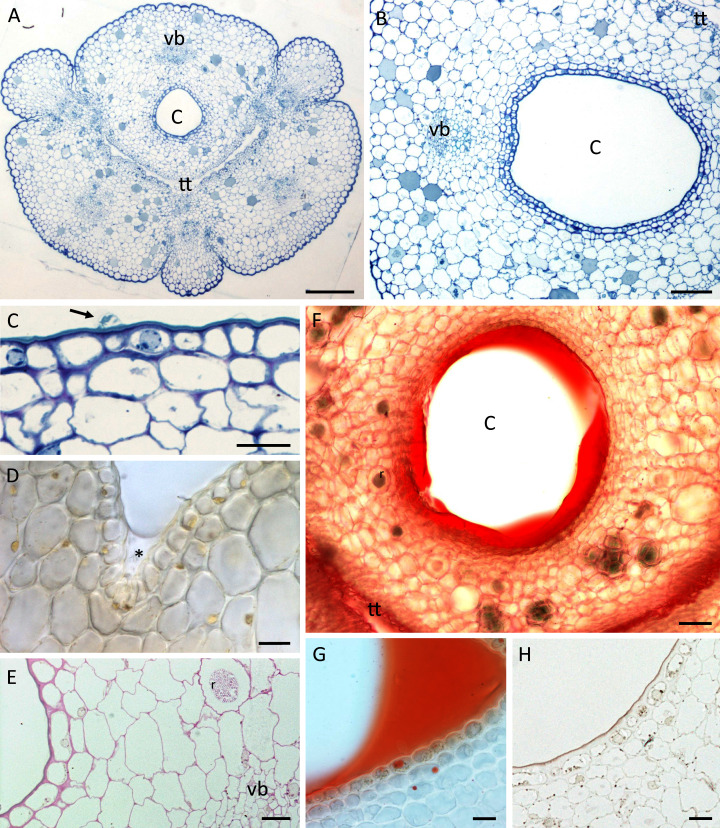
Nectary chamber and cuniculus of *B. whartoniana* (A, C, E–H) and *B. scandens* (B, D), transverse sections, LM. (A) Cuniculus and position of transmitting tract, stained with TBO; scale bar = 200 µm. (B) Cuniculus lined with smooth epidermis below which occur two layers of subepidermal cells, TBO; scale bar = 100 µm. (C) Nectary chamber. Epidermal cells with thick outer tangential (periclinal) cell walls and blistered cuticle (arrow), TBO; scale bar = 20 µm. (D) Cells lining nectary chamber stained with IKI. Note absence of starch and presence of colourless nectar (asterisk), hand-sectioned, non-fixed material; scale bar = 20 µm. (E) Section of cuniculus following PAS reaction. Note the presence of starch close to the vascular bundle; scale bar = 20 µm. (F) Cuniculus and nectar stained with Sudan IV, hand-sectioned, non-fixed material; scale bar = 50 µm. (G) Nectary chamber and nectar stained with Sudan IV, hand-sectioned, non-fixed material; scale bar = 20 µm. (H) Cells lining nectary chamber stained with SBB. Note thick outer tangential cell walls and cuticle; scale bar = 20 µm. C-cuniculus, r-raphides, tt-transmitting tract, vb-vascular bundle.

Both species had racemose inflorescences and structurally similar flowers (Figs. 1A–1D), with the labellum basally adnate to the column forming the nectary chamber (Figs. 2A, 2B). This chamber, at anthesis, in flowers of *B. whartoniana*, measured on average 1.4 mm × 3.2 mm (SD = 0.05 and 0.04, respectively), whereas in *B. scandens*, it measured on average 1.2 mm × 3.5 mm (SD = 0.07 and 0.04, respectively). In both species, at the level of the base of the perianth segments, the chamber became narrower, thus forming a tube-like cuniculus, which measured 23 mm (SD = 0.44) in length in *B. scandens*, and 14 mm (SD = 0.22) in length in *B. whartoniana*. The cuniculus runs alongside the transmitting tract and ovary (Figs. 1B, 2A, 2B and Figs. 3A, 3B, 3F). In both species, nectar was present in the nectary chamber (Figs. 2C, 2D) as well as in the narrow cuniculus (Figs. 2E and 3F). Small droplets of secretion were present in hand-sectioned buds, but larger ones were observed in open flowers (Fig. 2D). When viewed in daylight, the secreted nectar appeared clear and colourless (Fig. 2C), but when excited with UV-B light, it autofluoresced red (Fig. 2D, 2E). Sections stained with neutral red and with auramine O, and observed under UV-B and blue light, respectively, fluoresced yellow-green, indicating the presence of volatiles or lipid-like compounds within the nectar (Figs. 2F, 2G). Despite the obvious presence of secondary metabolites, the nectar had a sweet taste, and in *B. whartoniana*, gave a refractometer reading of 34.5%.

A single layered epidermis and 1–2 layers of small subepidermal parenchyma cells lined the nectary (Figs. 3A–3H). In both species, a single non-ramified vascular bundle ran through the innermost ground parenchyma in close juxtaposition to the nectary lumen (Figs. 3A, 3B, 3E). Starch was mainly absent from the epidermis and subepidermal parenchyma (Fig. 3D), but scant starch grains were observed in close proximity to the vascular bundles (Fig. 3E).

**Figure 4:**
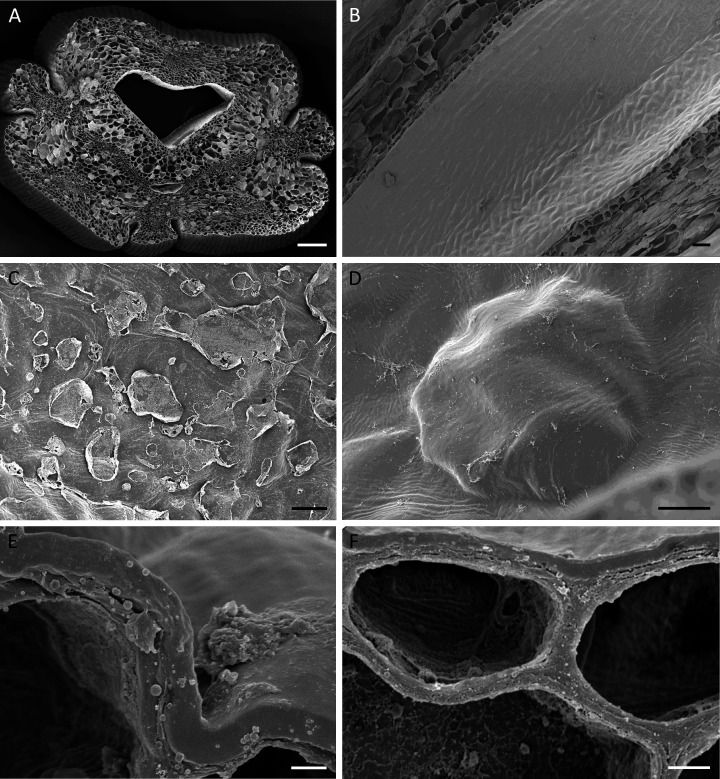
Cuniculus and nectary chamber of *B. scandens* (A, C, E, F) and *B. whartoniana* (B, D), SEM. (A) Transverse section of cuniculus lined with smooth epidermal cells; scale bar = 100 µm. (B) Longitudinal section of cuniculus showing surface of epidermis; scale bar = 30 µm. (C) Residues of surface secretion in nectary chamber; scale bar = 10 µm. (D)Distended cuticular blister on epidermal cell lining the nectary chamber; scale bar = 10 µm. (E) Detail of cell wall showing homogeneous structure of cuticle; scale bar = 1 µm. (F) Epidermal cells with thick outer tangential cell wall. Note lamellate structure of cellulosic part of the cell wall; scale bar = 2 µm.

In both species, the nectar stained with Sudan IV (Fig. 3F, 3G) confirming FM observations that lipids were present, but epidermal and parenchyma cells contained only a few lipid droplets (Figs. 3G, 3H). Scattered idioblasts with raphides were present in the parenchyma (Figs. 3E, 3F). The epidermal cells lining the nectary chamber and cuniculus were either flat or had slightly convex outer tangential cell walls (Figs. 3A–3H and Figs. 4A, 4B). Compared with the cell walls of deeply located ground parenchyma, their walls were thicker (Figs. 3B, 3C, 3E). The outer periclinal or tangential walls of epidermal cells were particularly thick and had a thick cuticle (Figs. 2F, 3C, 3H and Figs. 4E, 4F).

Light microscopy and SEM of the nectary chamber and cuniculus revealed residues of nectar and blistering of the cuticle, and some of these blisters had collapsed (Fig. 3C and Figs. 4C–4D). The cuticle overlying the epidermis had fine surface striations and no visible porosity (Fig. 4D).

**Figure 5:**
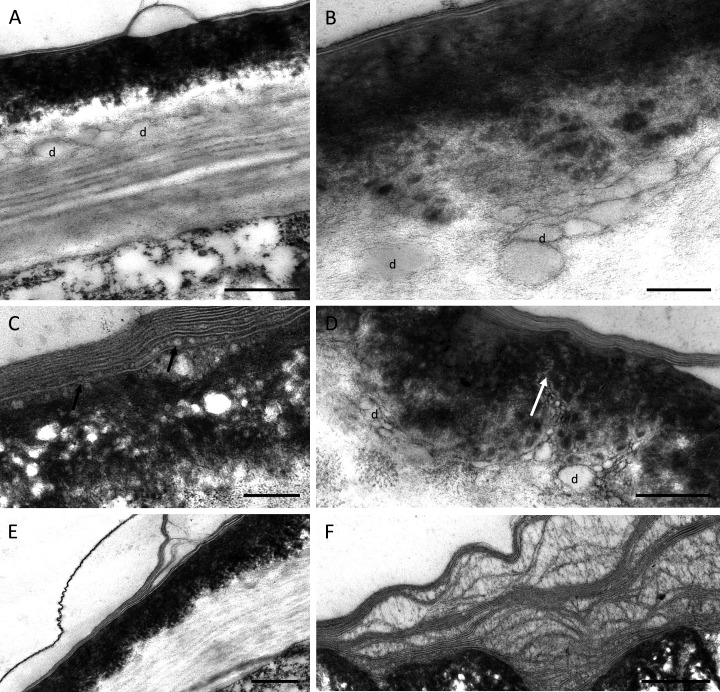
Outer cell wall in *B. whartoniana* (A, D, E) and *B. scandens* (B, C, F), TEM. (A) Droplets of lipid-like material located between cellulosic and electron-dense parts of the cell wall. Note blistering of cuticle; scale bar = 0.5 µm. (B). Detail of cellulosic part of the wall with associated droplets of lipid-like material; scale bar = 200 nm. (C) Lamellate cuticle with droplets visible between lamellae (arrows) and electron-dense part of the wall; scale bar = 200 nm. (D) Micro-channels (arrow) and lipid-like droplets in electron-dense part of the cell wall; scale bar = 0.5 µm. (E). Cuticle separating from the remaining part of the cell wall; scale bar = 0.5 µm. (F) Separated layers of cuticle with secreted surface material between striae; scale bar = 0.5 µm. d-lipid droplets.

TEM observations of nectary cells revealed that the outer tangential walls of epidermal cells lining the nectary chamber and cuniculus were multi-layered (Figs. 5A–5F) and their structure was similar in both investigated species. Commencing outwards from the plasmalemma, the cell wall was cellulosic with finely striate microfibrils (Figs. 5A, 5E). In *B. scandens*, this layer, on average, was 1.8 µm thick, whereas in *B. whartoniana*, it was, on average, 1.3 µm thick. The next layer was electron-dense and granular, and in both species, was on average, 0.5 µm thick (Figs. 5A–5F). In the outermost cellulosic layer of the wall, just beneath the electron-dense striae, occurred variously sized droplets of similar electron-density to the lipid-like material (Figs. 5A, 5B, 5D). Small droplets also accumulated in the micro-channels within the cuticular layer, and this was particularly evident in *B. whartoniana* (Fig. 5D), whereas in *B. scandens*, lipid-like droplets were dispersed throughout the electron-dense layer (Fig. 5C). Furthermore, in *B. whartoniana*, fine lamellate material ran vertically and was continuous with the lamellate cuticle (Fig. 5D). In both investigated species, TEM revealed the cuticle to be lamellate (Figs. 5A–5F), but this lamellate organization was not visible during SEM investigations (Figs. 4E, 4F). Globular material accumulated between the lamellae of the cuticle (Fig. 5C), and blisters or undulations were observed in this layer (Figs. 5A, 5E, 5F). In *B. whartoniana*, detachment of the whole laminar layer was more frequently observed (Fig. 5E) than in *B. scandens*.

**Figure 6:**
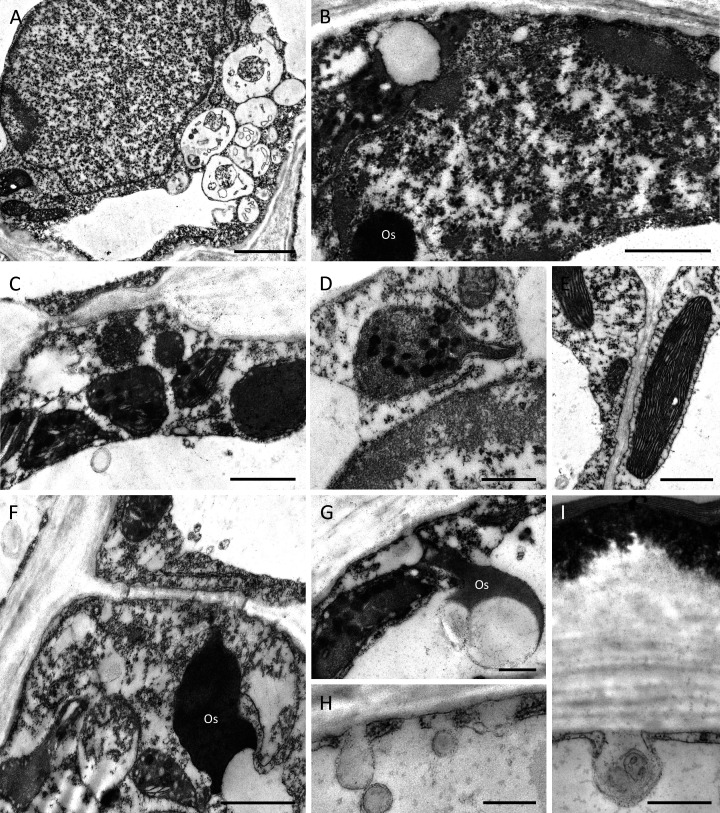
Ultrastructure of nectary cells of *B. scandens* (A, B, D, E, G, H, I) and *B. whartoniana* (C, F), TEM. (A) Ultrastructure of epidermal cell lining the cuniculus showing large nucleus, dense cytoplasm and several small vacuoles; scale bar = 2 µm. (B) Parietal, granular cytoplasm with plastid and electron-dense osmiophilic material; scale bar = 1 µm. (C) Starchless plastids with dense stroma and plastoglobuli. Plasmodesmata in primary pit-field; scale bar = 1 µm. (D) Detail of plastid with plastoglobuli; scale bar = 0.5 µm. (E) Chloroplast of subepidermal parenchyma cells containing very small starch grain; scale bar = 1 µm. (F) Cytoplasm of epidermal cell with plastids and osmiophilic material; scale bar = 1 µm. (G) Osmiophilic material and plastid in parietal cytoplasm; scale bar = 0.5 µm. (H, I) Secretory vesicles incorporated into periplasmic space; scale bar = 0.5 µm. Os-osmiophilic body.

Despite some slight differences in the structure of the outer tangential epidermal cell wall, the epidermal and subepidermal cells of both species had very similar ultrastructure (Figs. 6A–6I). The secretory cells possessed large nuclei and were vacuolate to various degrees. Some cells contained a single large, centrally positioned vacuole, whereas in others, sometimes in adjacent cells, several small vacuoles were present (Fig. 6A). The cytoplasm of epidermal cells predominantly contained plastids measuring 1–2 µm, each with an electron-dense stroma, few internal tubular lamellae and numerous spherical plastoglobuli (Figs. 6B–6D, 6F, 6G). Chloroplasts were present in the deeply located parenchyma (Fig. 6E). Plastids only occasionally contained small starch grains. Mitochondria and rough endoplasmic reticulum (RER) were distributed predominantly in the parietal cytoplasm (Figs. 6B, 6D, 6E), but smooth endoplasmic reticulum (SER) was seldom observed. Irregular osmiophilic bodies also occurred in the cytoplasm (Figs. 6B, 6F, 6G). Both epidermal and subepidermal cells were interconnected *via* plasmodesmata occurring in primary pit-fields (Figs. 6C, 6F), and secretory vesicles accumulated adjacent to cell walls and in the periplasmic space (Figs. 6H, 6I).

## Discussion

The flowers of the two investigated species of *Barkeria* had similar structure, and like the majority of Laeliinae, the labellum was adpressed to the column forming both a chamber and cuniculus, which together function as a nectary. Nectar was observed in both species, and this was secreted at the late bud stage and at the beginning of anthesis, despite previous reports by Soto Arenas & Solano-Gómez (2007) that *B. scandens* is nectarless. Like *Barkeria*, residues of nectar have also been observed in certain species of *Epidendrum* generally regarded as rewardless and deceptive. For example, close observations of cells lining the cuniculus of flowers of *E. capricornu*, *E. criniferum*, *E. pseudepidendrum*, *E. radicans*, and *E. xanthoianthinum* using LM, SEM and TEM revealed residues of secretion and demonstrated that such cells possess an organelle complement typical of secretory cells (Stpiczyńska et al., 2018). The discrepancy between the nectar observations made by Soto Arenas & Solano-Gómez (2007) on *Barkeria* flowers and the present results may perhaps be explained by the age of the flowers. The flowers investigated here were in bud stage or at the beginning of anthesis, whereas the flowers observed by the above authors may have been at advanced anthesis and, owing to reabsorption, may have appeared to be ‘empty’ of nectar. Furthermore, differences in the distribution of nectariferous and nectarless flowers at the population or individual plant level have also been reported for several natural populations of a range of different angiosperm species. Thus, differences in the nectariferous status of these plants may be related to the conservation of resources, whilst still ensuring the attraction of pollinators (Guimarães et al., 2018 and references therein; Pacini & Nepi, 2007; Thakar et al., 2003).

The nectar of both investigated species of *Barkeria* had a sweet taste, but also contained lipid-like compounds. Nectar is predominantly an aqueous solution of various sugars, but nearly all nectars investigated to date also contain a variety of additional components, including amino acids, organic acids, terpenes, alkaloids, flavonoids, phenolics, metal ions, oils, and proteins (Guimarães et al., 2018; Nepi, 2014; Nicolson & Thornburg, 2007 and references therein; Palmer-Young et al., 2019; Roy et al., 2017; Wiese et al., 2018). Secondary compounds in nectar may play a part in plant-animal interactions by repelling herbivores, regulating nectar ingestion, as well as having a significant role in defence against infection by pathogens. They also have an effect on the physiology of the nervous system of the visiting insect, and even promote muscle function during insect flight (Nepi, 2014). According to Palmer-Young et al. (2019), the ecological effects of secondary compounds are dose-dependent, and thus, heterogeneity across genotypes and populations is able to affect the evolution of floral traits and pollinator foraging ecology. The nectar of the investigated *Barkeria* spp. showed slight red autofluorescence when exposed to UV-B light. It would appear that this fluorescence does not act as a cue for visiting pollinators since nectar is concealed in the nectary chamber and cuniculus, and is therefore not visible at a distance. Chemical analysis of the secretion and detection of autofluorescent compounds, however, was not possible, but staining of the nectar and the nectary tissue with Sudan stains or neutral red indicated the presence of lipid-like material. Similar histochemical analysis results were also obtained for the resiniferous labellar surface material of various species of *Maxillaria* s.l. (Davies & Stpiczyńska, 2012, 2019; Stpiczyńska, Davies & Gregg, 2004), but unlike these, the flowers of *Barkeria* spp. investigated here seemingly lack fragrance, which is also a common feature of bird-pollinated flowers. That the presence of lipid-like compounds in nectar is an adaptation for the prevention of excessive evaporation of nectar in these xerophytic plants, as occurs in other angiosperms (Roy et al., 2017), cannot be ruled out, since both investigated species are exposed to high levels of solar radiation and water stress, in particular, during the dry season. Conversely, lipid is also common in the nectar of a range of non-orchidaceous Patagonian plants, occurring in 30–50% of the investigated species (Forcone, Galetto & Bernardello, 1997; Bernardello, Galetto & Forcone, 1999). It has been argued that caloric nectar is a good source of energy for pollinators under the extreme conditions found in this region. Furthermore, South African *Aloe castanea* has open, campanulate flowers, but although its nectar does not contain lipids, homeostasis is achieved on hot, sunny days by reabsorption of sugars (Nicolson & Nepi, 2005).

The ultrastructure of the nectary cell walls of *Barkeria* also appears to be an adaptation against drought-induced stress and for a xerophytic mode of life. The cell wall is very thick and multi-layered, and possesses a lamellate cuticle. Striae vary in their electron-density and contain micro-channels that facilitate the passage of nectar having both hydrophobic and hydrophilic components. Furthermore, the micro-channels in the cell wall contain variously sized vesicles of lipid-like material. Similar cell wall ultrastructure was noted in several species of *Epidendrum* (Stpiczyńska et al., 2018). Apoplastic transport of nectar in the thick cell walls could be an adaptation for reducing the evaporation of nectar constituents under dry conditions, as compared to transport *via* intercellular spaces. Thick, but permeable cellulosic cell walls resembling those of collenchyma cells were noted in nectaries of several epiphytic orchids (Stpiczyńska et al., 2018 and references therein). Nectar can therefore pass through the outer multi-layered tangential wall into the lumen of the nectary chamber or the cuniculus. Collenchyma has also been observed in several ornithophilous flowers, and is thought to reduce damage to the flower by the beaks of visiting birds (Stpiczyńska, Davies & Gregg, 2004). In other taxa that undergo dry-stress, *e.g.*, epiphytic root-less *Tillandsia juncea*, which is pollinated by hummingbirds, the nectary is septal, and the outer cell wall of the secretory epithelium is thick and has labyrinth-type ingrowths. Moreover, the nectar is enriched with lipids and protein (Mosti et al., 2013) that may be an adaptation for pollinator specificity, but also an adaptation for a xerophytic mode of life.

The proposed nectariferous status of the investigated *Barkeria* spp. is further strengthened by the ultrastructure of the epidermal cells lining the nectary chamber and cuniculus, as well as that of the underlying subepidermal cells. Such cells are typical of nectary cells in terms of their small size, their possession of a relatively large nucleus and dense cytoplasm, with numerous secretory vesicles that gather close to the cell wall. Small plastids with a dense stroma and electron-dense plastoglobuli closely resemble those present in nectary cells that secrete heterogeneous material, such as those of *Epidendrum* and *Maxillaria* (Davies & Stpiczyńska, 2019; Stpiczyńska, Davies & Gregg, 2004; Stpiczyńska et al., 2018), as well as those of certain non-orchidaceous genera *e.g., Jacaranda* (Bignoniaceae) (Guimarães et al., 2018). An atypical feature of the nectariferous cells of *Barkeria* was the absence of starch in plastids. Instead, starch grains were distributed in close proximity to the vascular bundles. In the majority of investigated taxa, nectariferous cells contain abundant starch, and this is also present in epidermal and subepidermal parenchyma cells, as well as in cells adjacent to the main vascular bundles supplying the nectary. The importance of starch has been widely reported for the floral secretory tissues (in particular, nectaries) of many taxa, including orchids, and it has been proposed that hydrolysis of starch reserves provides the metabolic energy for the secretory process, together with sugars for nectar production (Nicolson & Thornburg, 2007; Pacini & Nepi, 2007). Nevertheless, starchless plastids have also been observed in the nectariferous cells of *Gymnadenia conopsea* (Stpiczyńska & Matusiewicz, 2001), some species of *Epidendrum* (Stpiczyńska et al., 2018), as well as in *Fritillaria meleagris* (Liliaceae) (Stpiczyńska, Nepi & Zych, 2012).

In the investigated *Barkeria* species, secretory parenchyma cells contained plastids having an electron-dense stroma, indicating that they might be engaged in the synthesis of various secondary metabolites that are frequently present in nectar.

## Conclusions

Flowers of *B. whartoniana* and *B. scandens* are adapted to nectar secretion in extremely dry conditions. Robust secretory tissue, thick cell walls, and lipid-like nectar components limit nectar evaporation. Both investigated *Barkeria* spp. are endangered. It appears that the overall threat involves a number of environmental factors. These include the availability of suitable host trees, pollinator limitation (Angulo, Ruiz-Sanchez & Sosa, 2012), felling and the impact of agriculture on suitable habitats, and the reckless collection of plants. The ability of *B. scandens* to switch from entomophily to ornithophily (Warford, 1993), however, indicates that this species is already able to adapt to certain changes in its environment. Finally, the potential capacity for *B. scandens* to evolve in this way is clearly closely related to the structure of its flower and to the composition of the nectar it produces.

## Supplemental Information

10.7717/peerj.11874/supp-1 Supplemental Information 1 Nectary size of *Barkeria scandens* and *B. whartoniana*. Data indicates measurement of nectary chamber and cuniculus in five flowers

## Additional Information and Declarations

### Data Availability

The following information was supplied regarding data availability: The raw measurements are available in the Supplemental File.
